# Comprehensive characterization of chemotherapeutic efficacy on metastases in the established gastric neuroendocrine cancer patient derived xenograft model

**DOI:** 10.18632/oncotarget.3712

**Published:** 2015-03-30

**Authors:** Jiahua Jiang, Daisy Dandan Wang, Mengmeng Yang, Dawei Chen, Liang Pang, Sheng Guo, Jie Cai, Jean-Pierre Wery, Linda Li, Henry Qixiang Li, Peter Ping Lin

**Affiliations:** ^1^ Crown Bioscience, Santa Clara, California, USA; ^2^ Cytelligen, San Diego, California, USA; ^3^ State Key Laboratory of Natural and Biomimetic Drugs, Peking University, Beijing, China

**Keywords:** CTC subtypes, metastatic PDX, iFISH, chemoresistance, *in situ* phenotyping and karyotyping

## Abstract

The HuPrime^®^ human gastric neuroendocrine carcinoma derived xenograft model GA0087 was established in this study. GA0087 PDX model showed high gene expression of vascular endothelial growth factors (VEGF)-A and B, and high potential of lung metastasis. Circulating tumor cells (CTCs) with either large or small size, circulating tumor microemboli (CTM) and lung metastatic lesions were detected in GA0087 PDX mice. The number of CTC correlated to the number of metastatic nodules in lung. Both primary tumor growth and metastasis in terms of the number of dynamically monitored CTCs and metastatic nodules were effectively suppressed by Cisplatin. Diverse subtypes of CTCs in the context of sensitivity to Cisplatin were specifically identified by subtraction enrichment (SE) integrated with *in situ* Phenotyping of cytokeratin 18 (CK18) and Karyotyping of chromosome 8 (*in situ* PK CTC by CK-iFISH). All the CK18-/diploid and majority of CK18+/diploid CTC subtypes were chemosensitive, whereas a higher percentage of CK18+/multiploid subtype of CTC were Cisplatin-insensitive. Combined histopathological examination of metastatic lesion and *in situ* PK CTC in a metastatic PDX (mPDX) tumor model are of particular significance, and may provide an unique and robust platform for cancer research as well as pre-clinical evaluation of therapeutic efficacy of new anti-cancer drugs.

## INTRODUCTION

Gastric cancer (GC) leads to the 3rd cancer-related mortality worldwide [1]. Overall 5-year survival rate of GC patients is as low as only 20% due to either chemoinsensitivity and/or early development of chemoresistance of GC to therapeutic agents [2, 3].

Advantages of novel preclinical patient derived xenograft (PDX) models over the traditional tumor animal models established with cancer cell lines for development of anti-cancer drugs have been reported [4-7]. Unlike cell lines showing great genetic divergence comparing to the primary tumors in cancer patients, PDX models closely recapitulate the heterogeneity of patients' primary tumors and possess biological stability of gene-expression and mutational status, etc. Such superiorities offer the promise that PDX models will predict new anti-cancer drug efficacy including both sensitivity as well as resistance more reliably than cancer cell lines [7, 8]. However, PDX model with high metastasizing potential has been reported rarely.

Angiogenesis is essential for tumor growth and metastasis and is controlled by angiogenic factors. One of the important angiogenic factors is vascular endothelial growth factor-A (VEGF-A) produced by tumor cells [9]. VEGF-A facilitates hyperpermeability and macromolecular transvascular transport [10]. In particular, it has been reported that VEGF-A expression correlates with distant hematogenous metastases in gastric carcinoma patients [11, 12].

The clinical significance of circulating tumor cells (CTCs) and circulating tumor microemboli (CTM, a cluster of 2 or more CTCs) [13, 14] in tumor metastasis of various cancer types [15] including gastric [16] and lung [17] carcinomas has been documented elsewhere. The American Society of Clinical Oncology (ASCO) has recently accepted quantification of CTC as a novel breast cancer biomarker [18]. Currently, the conventional CTC detection methodology relies on expression of both EpCAM for isolation, and intracellular cytokeratins (CK) for identification [19]. However, it has been recognized that clinical application of such strategy could be significantly limited due to highly heterogeneous and dynamic expression of EpCAM among different cancer cells [20, 21]. Moreover, down-regulation or loss of both EpCAM and CK during epithelial-mesenchymal transition (EMT), a key process for generation and dissemination of CTCs via the circulation, has been reported by others [20-22]. In addition, because EpCAM-related intracellular signaling pathways in cancer cells can be activated following binding of anti-EpCAM to neoplastic cells, it is not surprising that subsequent analysis of CTCs perturbed by anti-EpCAM may result in post-isolation artifacts [20, 23, 24].

A novel combined cellular and molecular approach of integrated subtraction enrichment (SE) and immunostaining-FISH (i•FISH^®^) to detect and characterize CTCs has recently been reported [16]. SE-i•FISH^®^ is able to enrich and detect different subtypes of CTC regardless of caner types, CTC size variation [25] and CK or EpCAM expression. Obtained CTCs, free from hypotonic damage [26] and anti-EpCAM purturbing, are suitable for primary tumor cell culture and a series of subsequent studies including gene mutation analysis performed on the individual CTC [27] as well as establishing tumorigenic CTC- or CTC subtype-derived xenograft mouse models (CDXs) which could mirror the donor patient's response to chemotherapy [28].

The HuPrime^®^ PDX mouse model, GA0087, derived from human gastric neuroendocrine carcinoma has been successfully established in this study. The GA0087 model had a high gene expression of VEGF-A and B and demonstrated a high metastasizing potential showing both lung metastatic lesion in 88% of GA0087 mice, and detectable CTC in 86% of those metastatic lesion positive mice. Studies of dynamic status of CTCs demonstrated the existence of hematogenous dissemination waves of CTC (or “CTC waves”) in non-treated metastatic GA0087 PDX mice. Both primary tumor growth and tumor metastasis in terms of the number of CTCs and metastatic nodules in lung were suppressed following administration of chemotherapeutic agent cisplatin. Moreover, in accordance with our previous study demonstrating that chromosomal aneuploidy in CTCs correlates to either intrinsic or the acquired cisplatin resistance in gastric cancer patients [16], different subtypes of CTC classified upon their chromosome 8 ploidy and CK18 expression examined by CK-i•FISH^®^ were found to have distinct diverse sensitivities to cisplatin in GA0087 PDX mice. Pre-clinical therapeutic drug efficacy evaluated by the integrated subtraction enrichment (SE)-i•FISH^®^ on the additional PDX models established from melanoma, colorectal, non-small cell lung cancer (NSCLC) and hepatocellular (HCC) carcinoma patients are currently under our investigation.

In contrast to conventional measuring tumor mass and enumerating metastatic nodules alone, comprehensive characterization of metastasis performed by the combined immuno-histopathological examination of metastatic lesion and *in situ* phenotypic and karyotypic characterization of CTC (*in situ* PK CTC) in a metastatic PDX (mPDX) tumor model are of particular significance for cancer research as well as development of new anti-cancer therapeutic strategies and agents.

## RESULTS

### Histopathological characterization and genomic profiling of the metastatic HuPrime^®^ PDX model GA0087

**Figure 1:**
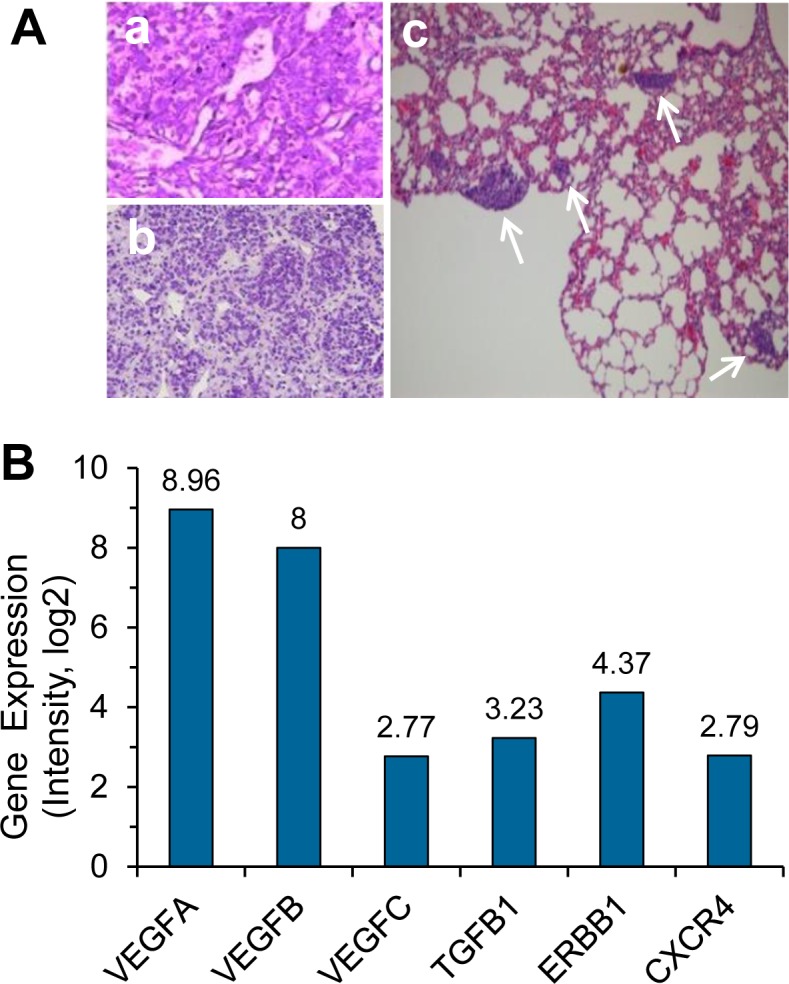
Histopathological and genomic characterization of the metastatic HuPrime^®^ PDX model GA0087 (**A**) Histopathological examination shows images of (a) donor patient's primary gastric neuroendocrine tumor tissue, (b) re-engrafting, and (c) metastatic lesions in the lung of GA0087 mice indicated by white arrows. (**B**) Genomic profiling shows that GA0087 model has a higher gene expression of VEGF-A and VEGF-B, comparing to VEGF-C, TGFB1, ERBB1 and CXCR4, respectively.

Diagnosis of the donor patient's primary gastric neuroendocrine cancer was confirmed by histopathological biopsy as shown in Figure 1A-a. Tumor was surgically removed from the patient, and the small 3×3×3 mm^3^ freshly dissected tumor tissue was subcutaneously (Sub-Q) engrafted on the immunocompromised nude mice, followed by serial re-engraftings when each growth reached 500-700 mm^3^ from P1 to P10. The tumor tissue of GA0087 P8 revealed in Figure 1A-b was applied for Sub-Q implantation on different mice in this study. Metastatic lesions in the lung of GA0087 mice were shown in Figure 1A-c, indicating that the established GA0087 was an authentic metastatic PDX model.

As demonstrated in Figure 1B, subsequent gene expression analysis performed on the frozen tissue cells of mice indicated that cells in GA0087 mice particularly had a high gene expression of VEGF-A and VEGF-B, which were 2-3 times higher than other examined genes including VEGF-C, TGFB1, ERBB1 and CXCR4.

Additional genetic and genomic profiling revealed that gene of cells in GA0087 mice was wild type for EGFR, ERK, BRAF, KRAS, PI3K, and c-MET (data not shown).

### *in situ* phenotypic and karyotypic identification and characterization of CTCs by CK-iFISH

**Figure 2:**
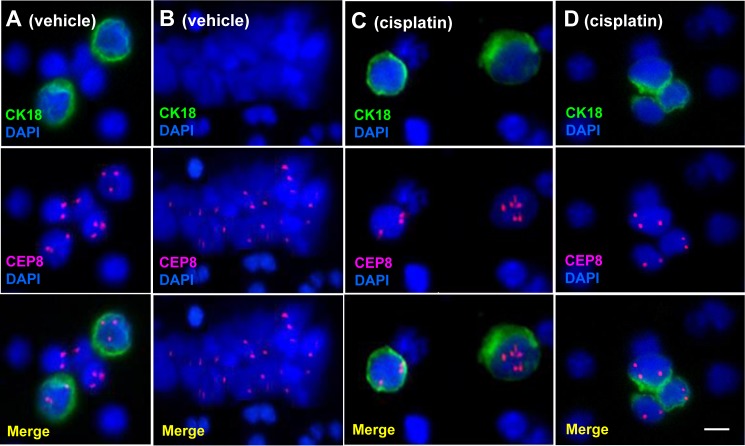
*in situ* Phenotypic and karyotypic detection and characterization of CTCs by CK-iFISH (**A**) A CTM consists of 2 large CK18+ triploid and 3 small CK18- heteroploid CTCs. (**B**) An identified CK18- CTM consists of small tumor cells. Tumor cells in (A) and (B) were detected in vehicle mice. (**C**) Large CK18+ triploid and multiploid (≥5 copies of chromosome 8) CTCs are demonstrated. (**D**) A CTM consists of 2 small diploid and 1 large triploid CTCs with heterogeneous expression of CK18. Tumor cells in (C) and (D) are from cisplatin treated mice. Neither anti-human CK18 nor CEP8 shows positive signal in mouse WBCs. Bar = 5 μm.

*in situ* PK CTCs and CTM enriched from 200 μl of GA0087 murine blood was performed by CK-iFISH. Mouse blood samples were subjected to subtraction enrichment (SE). Enriched CTCs on the coated CTC slides were hybridized with the human chromosome 8 centromere probe and immunostained with anti-CK18 antibody, followed by image acquiring and analysis. Demonstrated in Figure 2A, a CTM consisting of diverse subtypes of CTC including 2 large cells of CK18+/triploid, 3 small cells of CK18- respectively with monoploid, diploid and triploid chromosome 8 was shown in a vehicle control mouse. Figure 2B showed a CK18- CTM consisting of small tumor cells observed in a vehicle mouse. In cisplatin-treated GA0087 mice, large CK18+ triploid and multiploid (≥5 copies of chromosome 8) CTCs were demonstrated in Figure 2C. A CTM consisting of 2 diploid and 1 triploid tumor cells with heterogeneous expression of CK18 was revealed in Figure 2D, 2 diploid CTCs in this CTM were as small as mouse WBCs, whereas the triploid CTC was large. Mouse WBCs were negative for both anti-human CK18 staining and human CEP8 iFISH.

### Cisplatin suppresses primary tumor growth and extends survival time of GA0087 PDX mice

**Figure 3:**
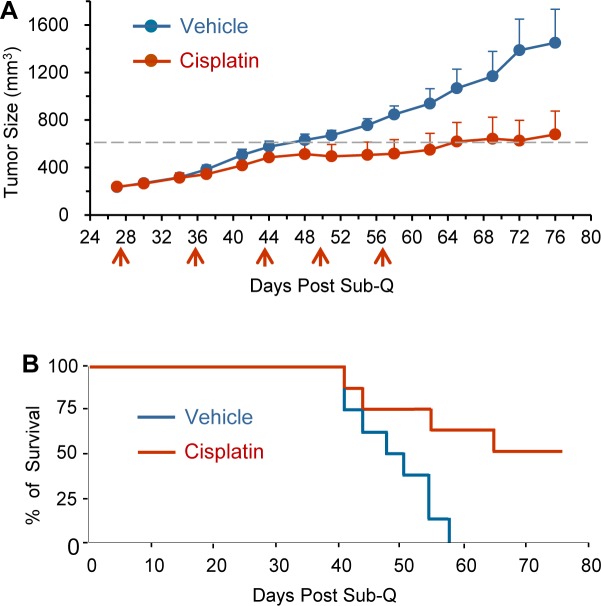
Cisplatin suppresses primary tumor growth and extends survival time of GA0087 mice (**A**) Cisplatin suppresses primary tumor growth. Comparing to vehicle mice *(n* = 8), cisplatin treated GA0087 mice (*n* = 8) show 20 days delayed to reach the median tumor growth size of 600 mm^3^ (grey dash line), *P* < 0.05. At the end of the experiment on Day 76, T/C value = 36%, *P* < 0.05. Administration of cisplatin is indicated by red arrows. (**B**) Results of Mantel-Haenszel Test on Time-to-Event (Event: the medium tumor growth size of 600 mm^3^) resembling the survival time, shows a hazard ratio (HR) of 5.374 with 95% CI of 1.431-20.18, indicating cisplatin treated mice have a longer survival time compared to that of vehicle mice.

Intravenous administration of cisplatin (treated group) or 0.9% NaCl (vehicle group) was performed for total of 5 times at the indicated time intervals (red arrows) on 8 of randomly selected GA0087 PDX mice from each group. Initial cisplatin treatment started on Day 27 since subcutaneous (Sub-Q) implantation of tumor tissue on Day 1. Cisplatin efficacy on primary tumor growth and survival time was investigated on GA0087 mice. Figure 3A showed that tumor growth for the cisplatin treated mice was 20 days delayed to reach the median tumor growth value of 600 mm^3^ (grey dash line), compared to that of the vehicle control group. The difference between the two groups was statistically significant (*P* < 0.05). At the completion of the experiment on Day 76, the average tumor size for cisplatin and vehicle group was 678 mm^3^ (initial = 238 mm^3^) and 1450 mm^3^ (initial = 235 mm^3^), respectively. The T/C value = ΔT/ΔC × 100% = (678-238)/(1450-235) × 100% = 36%, *P* < 0.05, suggesting that primary tumor growth in GA0087 PDX mice was significantly suppressed by cisplatin.

Each vehicle and cisplatin treated group had 2 tumor free-mice as a negative control. None of those negative control mice was found to have primary tumor.

Results of Time-to-Event analysis (Event: the medium tumor growth size of 600 mm^3^) performed by log-rank (Mantel-Haenszel) test (Figure 3B) showed a hazard ratio (HR) of 5.374 with 95% Confidence Interval (95% CI) of 1.431-20.18. Survival analysis indicated a longer survival time in cisplatin treated mice compared to that of vehicle group, suggesting that cisplatin had an effective anti-tumor activity in the established PDX model GA0087.

### Cisplatin suppresses tumor metastases in PDX mice

Cisplatin efficacy on metastases was quantitatively examined on both CTC and lung metastatic lesion in 8 mice of each treated or vehicle group. The number of CTC in cisplatin treated or vehicle GA0087 mice was continuously enumerated during the study, and the lung metastatic nodules were quantified at the completion of the experiment on Day 76.

Histopathological examination showed that 7 out of 8 mice in each treated or vehicle group developed metastatic lesions in lung. Five out of those 7 lesion positive vehicle-mice were found to have detectable CTCs, whereas 7 out of those 7 lesion positive treated-mice were CTC positive. The mice, which didn't show metastatic lesion in each group (1 out of 8), were not found CTC positive either. Neither metastatic lesion nor CTC was observed in 2 of tumor free-negative control mice in each vehicle and treated group.

**Figure 4:**
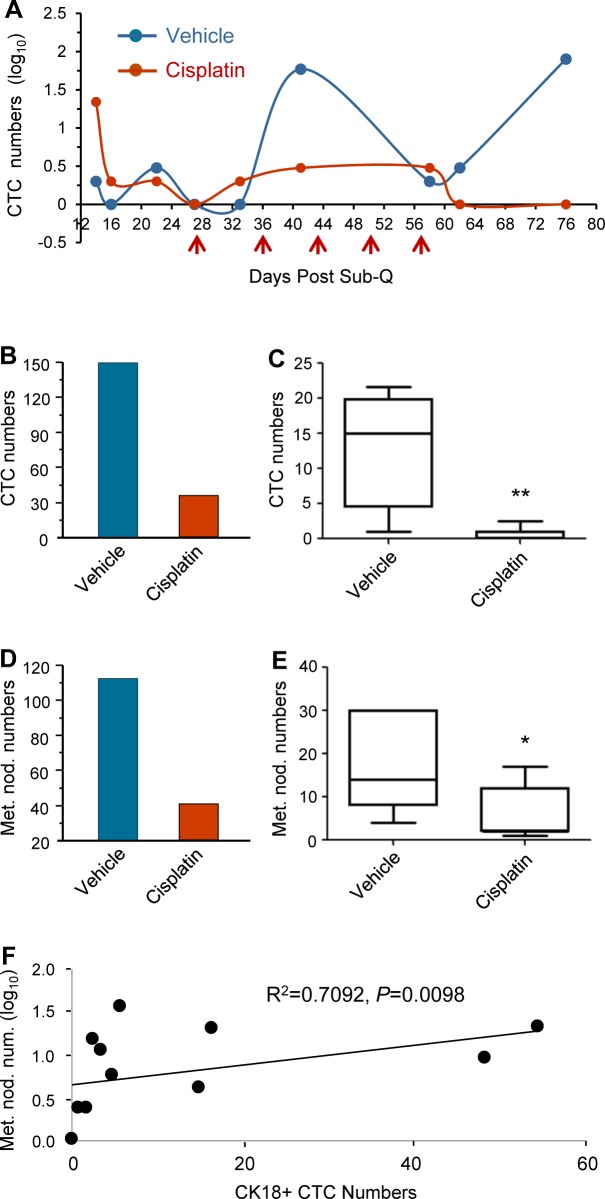
Cisplatin suppresses tumor metastases in GA0087 mice (**A**) Cisplatin decreases the number of CTC. Comparing to CTCs in non-treated vehicle mice, the number of CTCs in cisplatin treated mice is significantly lower following 5 times of cisplatin treatment (red arrows). “CTC waves” peaked at Day 22, 41 and 76 are observed in vehicle mice. (**B** and **C**) Quantification and statistical analysis of CTCs. The total number of 150 CTCs in vehicle mice (B, blue, *n* = 5) with the median of 15 shown in (C) are demonstrated, compared to total of 35 CTCs in treated mice (B, red, *n* = 7) with the median of 1 revealed in (C). The difference of CTC numbers between the vehicle and treated group is statistically very significant, **P < 0.01. (**D** and **E**) Quantification and statistical analysis of metastatic lesion. The total number of metastatic nodules in vehicle mice is 113 (D, blue, *n* = 7) with the median of 14 shown in (E), compared to the total number of 41 metastatic nodules in treated mice (D, red, *n* = 7) with the median of 2 demonstrated in (E). The difference of metastatic nodule numbers between the 2 groups is statistically significant, *P < 0.05. (**F**) Results of Spearman Rank Correlation Test indicate the number of metastatic nodule in lung statistically correlates to the number of CK18+ CTC with 95% CI of 0.2097 to 0.9152, Spearman's rho (R^2^) = 0.7092, *P* < 0.01.

Dynamic dissemination of CTC in both treated and vehicle GA0087 mice was monitored and shown in Figure 4A. Two hundred μl of blood were serially collected from Day 14 to Day 76 with a total of 9 collections, followed by subjection to SE-iFISH for CTC detection. Administration of cisplatin was performed at the indicated time intervals (red arrows). Results of Figure 4A demonstrated that in both treated and vehicle mice, CTCs could be detected as early as 2 weeks since Sub-Q implantation on Day 1. Comparing to CTCs in vehicle mice, the number of CTC in the treated mice was significantly lower, whereas a few CTCs (8 cells) were still detected on Day 62 following 5 times administration of cisplatin initiated on Day 27. All the CTCs were eventually eliminated in the treated mice at the completion of the experiment on Day 76. Obtained results indicated that majority of the disseminated tumor cells were sensitive to cisplatin, but there were few cisplatin-insensitive CTCs existed. Monitoring of CTCs in non-treated vehicle mice showed existence of “CTC waves” peaked at Day 22, 41 and 76, respectively.

Additional analysis was performed to further quantitatively and statistically characterize CTCs in vehicle and cisplatin treated mice. Revealed in Figure 4B/4C, total CTC numbers in 5 of CTC positive vehicle mice was 150 (B, blue column, *n* = 5) with a median of 15 (Min 1, Max 22) as shown in Figure 4C. With respect to those cisplatin treated mice, CTCs were detected in all of 7 mice, and total of 35 CTCs (B, red column, *n* = 7) with a median of 1 (Min 0, Max 2.5) (Figure 4C) were identified. The difference of total CTC numbers between the vehicle and the treated group was statistically very significant (**P < 0.01).

Quantitative and statistical analysis of metastatic nodules shown in Figure 4D/4E demonstrated the total number of metastatic nodules in 7 vehicle-mice was 113 (D, blue column, *n* = 7) with a median of 14 (Min 4, Max 30) as revealed in Figure 4E. Total of 41 metastatic nodules were identified in 7 treated-mice (D, red column, *n* = 7) with a median of 2 (Min 1, Max 17) (Figure 4E). The difference of metastatic nodule numbers between the vehicle and treated group was statistically significant (*P < 0.05).

Sperman Rank Test was performed to examine if there was a correlation between CTC numbers and metastatic nodule numbers. Demonstrated in Figure 4F, the results of Spearman's rho analysis showed (R^2^) = 0.709 with 95% CI of 0.2097 to 0.9152, *P* < 0.01, indicating that there was a statistically very significant numerical correlation between the lung metastatic nodule numbers and the CK18+ CTC numbers in GA0087 PDX mice.

Obtained results indicated that metastases in terms of the enumerated CTCs and metastatic nodules were effectively suppressed by cisplatin in GA0087 PDX mice, suggesting that quantification of both CTC and metastatic lesion is applicable for efficient pre-clinical evaluation of anti-cancer drug efficacy in metastatic PDX (mPDX) mice.

### Diverse subtypes of CTC possess different sensitivities to cisplatin

Some CTCs were detected on Day 62 following administration of cisplatin for 5 times or 5 weeks, suggesting existence of cisplatin-insensitive CTCs in the treated GA0087 mice. We took the advantage of *in situ* PK CTC performed by CK-iFISH to identify and characterize CTC subtypes for the purpose of pinpointing the specific subtype insensitive to cisplatin. CTCs subtypes were classified upon their CK18 expression and chromosome 8 ploidy in this study.

**Figure 5:**
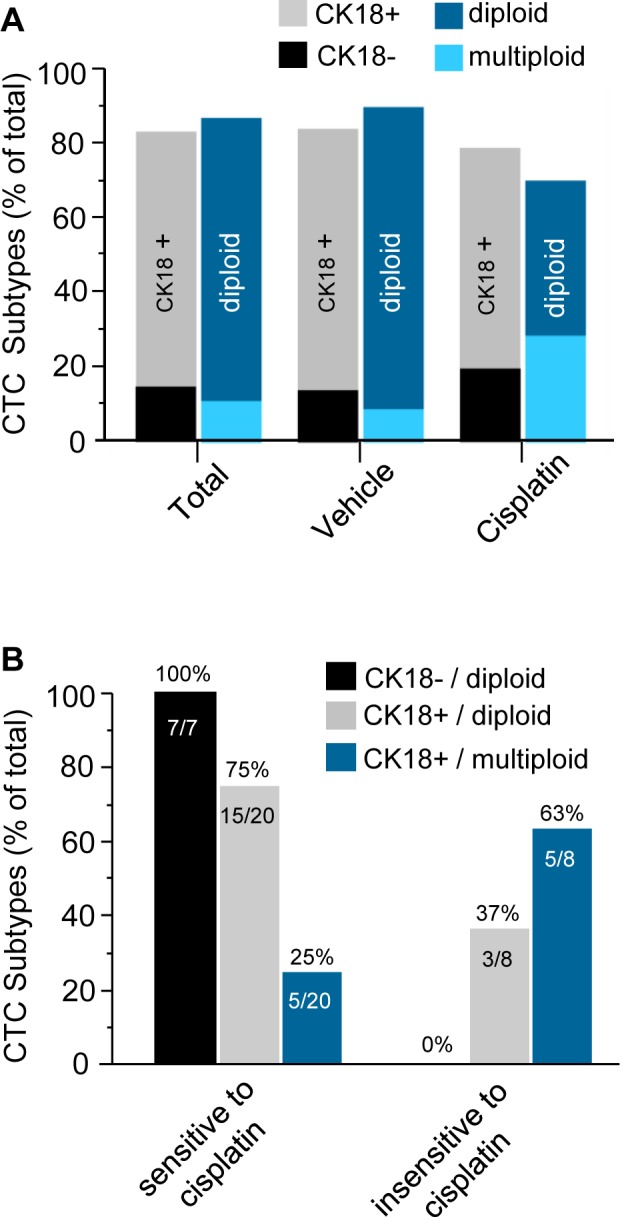
Diverse CTC subtypes possess variable sensitivities to cisplatin (**A**) Quantitative analysis of CTC subtypes. Among total of 185 CTCs detected in both vehicle and treated mice, 84% of CTCs are CK18+, and the rest of 16% are CK18-; 88% are diploid, and remainng12% are multiploid, respectively. Among 150 CTCs in the vehicle group, 85% are CK18+, and the rest of 15% are CK18-; 91% of those CTCs are diploid, and remaining 9% are multiploid. Regarding 35 CTCs identified in the cisplatin treated mice, 80% are CK18+, and the other 20% are CK18-; 71% are diploid and rest 29% are multiploid. (**B**) Characterization of CTC subtypes either sensitive or insensitive to cisplatin in the treated group of mice. Among 27 of chemosensitive CTCs detected only prior to Day 62, there are both CK18- and CK18+ tumor cells. All of CK18- CTCs are sensitive to cisplatin, and they are all diploid (100%); for those CK18+ cisplatin-sensitive CTCs, 75% of them are diploid, and remaining 25% are multiploid. Regarding 8 of cisplatin-insensitive CTCs detected on Day 62, none of them (0%) are CK18- but all CK18+. Majority (63%) of those chemoinsensitive CTCs are CK18+/multiploid, and the rest 37% are CK18+/diploid.

First of all the ratio of different CTC subtypes among the total CTC population and those in vehicle or cisplatin treated mice was analyzed. Demonstrated in Figure 5A, among the total of 185 CTCs including 150 in vehicle and 35 in cisplatin treated mice, 84% (155 out of 185) were CK18 positive, and the remaining 16% (30 out of 185) were CK18 negative; 88% of the total (162 out of 185) were diploid, and the rest 12% (23 out of 185) were triploid or multiploid. Further analysis of 150 CTCs in vehicle mice revealed that 85% (117 out of 150) were CK18 positive, and the remaining 15% (23 out 150) were CK18 negative. Ninety one percent (137 out of 150) of those CTCs were diploid, and the remaining 9% (13 out of 150) were multiploid with the majority of trisomy. Of the total of 35 CTCs detected in cisplatin treated mice, 80% (28 out of 35) were CK18 positive, and the remaining 20% (7 out of 35) were CK18 negative; 71% (25 out of 35) of these CTCs were diploid, and 29% (10 out of 35) were multiploid with the majority of trisomy.

Additional detailed analysis of CTC subtypes with diverse sensitivities to cisplatin was summarized in Table 1. Twenty three out of the 150 CTCs in vehicle mice were CK18 negative, and they were diploid (15%, 23 out of 150 total CTCs). The remaining 127 CTCs were CK18 positive, including 114 diploid (76%, 114 out 150) and 13 multiploid (9%, 13 out of 150). Among 35 CTCs in the treated mice, 8 cells could be detected on Day 62 following administration of cisplatin for 5 times, and those cells were cisplatin-insensitive-CTCs, whereas other 27 detectable CTCs were eliminated by cisplatin prior to Day 62, and they were defined as cisplatin-sensitive-CTCs in this study. Regarding 27 cisplatin-sensitive-CTCs consisting of 7 CK18- and 20 CK18+ cells, 7 CK18- CTCs were all diploid (100%, 7 out of 7), and remaining 20 CK18+ cells had 15 diploid (75%, 15 out of 20) as well as 5 multiploid CTCs (25%, 5 out of 20). With respect to the 8 cisplatin-insensitive-CTCs, all were CK18 positive, including 37% (3 out of 8) diploid and 63% (5 out of 8) multiploid cells.

Further analysis of those 35 CTCs in the treated mice shown in Figure 5B revealed that all of the CK18 negative CTCs (100%) were diploid and chemosensitive. However, CK18 positive CTCs were either sensitive or insensitive to cisplatin depending on their chromosomal ploidy. Taken together, the CK18+/diploid subtype constituted the main population (75%) of chemosensitive CTCs, whereas the CK18+/multiploid subtype constituted the majority (63%) of cisplatin-insensitive CTCs.

**Table 1:** *in situ* Phenotypic and Karyotypic Characterization of CTC Subtypes and Their Diverse Sensitivities to Cisplatin

CTCs	CK18 -	CK18 +	
	diploid	diploid	multiploid
Vehicle	15% (23/150)	76% (114/150)	9% (13/150)
Sensitive to cisplatin	26% (7/27 total) 100% (7/7 CK18-)	55.5% (15/27 total) 75% (15/20 CK18+)	18.5% (5/27 total) 25% (5/20 CK18+)
Insensitive to cisplatin	0	37% (3/8)	63% (5/8)

## DISCUSSION

The predictability of drug efficacy in mouse models employing human tumor cell lines has major limitations that include frequent genetic drift *in vitro* with cell passages, thus their resemblance to actual human tumor specimens is usually significantly different. Pre-clinical PDX models have been utilized for testing the efficacy of experimental anti-cancer drugs [7], particularly because they appear to maintain strong genetic resemblance to the parental tumor [5-8, 28]. However, one limitation of PDX models is the failure of these tumors to metastasize [6, 7]. Gene-expression profiling of the gastric neuroendocrine carcinoma PDX model GA0087 established in this study revealed particularly high gene expression of VEGF-A and B, both of which have been recognized as having a significant role in early tumor development [9] and angiogenesis [10]. Moreover, VEGF-A has been reported to correlate to distant hematogenous metastasis in gastric cancer patients [11, 12]. The high gene expression of VEGF-A in GA0087 PDX mice is consistent with our current observations showing that GA0087 PDX model has high metastasizing potential to lung.

Although metastases were not found in the donor GC patient (TNM Staging II, T3N1M0) by the time when surgical resection was performed, the tumor did metastasize in the PDX model GA0087, suggesting that additional host factors prior to tumor resection in patients, including CTC subtype status and/or VEGF-A expression, etc. could be the more significant “predictor” with respect to establishing a metastatic PDX (mPDX) model. Moreover, the PDX mouse model may recapitulate events that will occur in the future in the patient. Given the reality that CTC numbers do not always correlate with the conventional TNM staging [17, 29], correlation of CTC subtype status in donor patients with metastasizing potential of PDX mice is currently under our investigation in large cohorts of cancer patients at different TNM stages.

Application of CTC to evaluate therapeutic drug efficacy in a mPDX model has been rarely reported. The conventional CTC detection technology is restricted to enumeration of only both EpCAM and cytokeratin (CK) positive CTCs [19]. However, down-regulation or loss of both EpCAM and CK were reported in EMT CTC [20], such inherited property of tumor cell may result in failure to detect significant amount of CTCs by the conventional strategy [30, 31]. In this study, we took advantage of SE-i•FISH^®^ [16] to enrich, detect and characterize different subtypes of CTC for the purpose of evaluating chemotherapeutic efficacy of cisplatin in mPDX mice. Both CTM and individual CTCs with different sizes varying from that as small as WBCs to larger cells in GA0087 mice could be efficiently detected.

**Figure 6:**
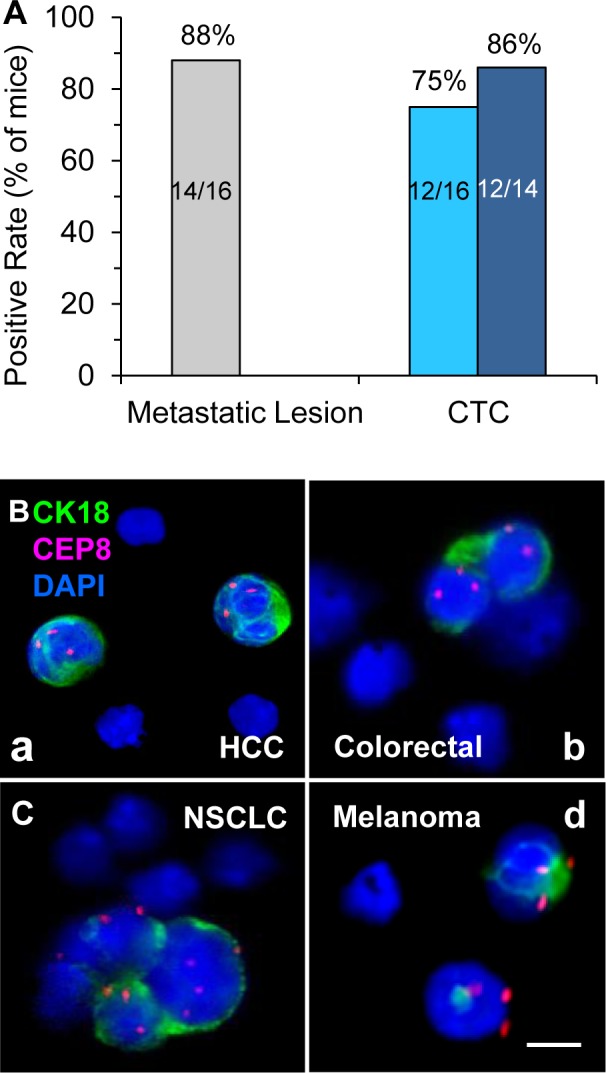
Analysis of metastatic potential of GA0087 mice and *in situ* PK CTC in different PDX models (**A**) Analysis of GA0087metastasizing potential. Fourteen out of total 16 mice (88%, grey) have metastatic lesions in lung, and 12 out of those total 16 mice (75%, light blue), or 12 out of 14 metastatic lesion positive mice (86%, dark blue) show CTC positive. (**B**) *in situ* PK CTC in different PDX models. (B-a) Two of the CTCs enriched from a HCC PDX mouse (LIM612) show CK18+ and chromosome 8 triploid. (B-b) Diploid and triploid CK18+ colorectal cancer CTCs which are as small as mouse WBC (mWBC) are identified in the PDX mouse (CR004). (B-c) In a NSCLC PDX model (LUM2509), a CTM consisting of 4 tumor cells with variable sizes of larger or similar to that of mWBCs shows highly heterogeneous expression of CK18 and polysomy chromosome 8 from mono- to tetraploid. (B-d) Two triploid melanoma CTCs with positive CK18 expression are detected in a melanoma PDX mouse (ME1154). mWBCs are negative for both human CK18 immunofluorescent staining and human CEP8 FISH. Bar = 5 μm.

High metastasizing potential in terms of the percentage of CTC and/or metastatic lesion positivity in GA0087 mice was demonstrated in Figure 6A, showing that 7 out of 8 treated or vehicle mice (7 out of 8 or 14 out of total 16, 88%) were found metastatic lesion positive in lung, and 12 of those total 16 mice showed detectable CTC (12/16, 75%). Among those 14 lesion positive mice, CTCs were found in 12 mice including all the 7 treated and 5 of untreated vehicle mice, the positive rate was 86% (12 out of 14). In the current study, tumor heterogeneity of variable metastasizing potential was observed in the control group. Two vehicle mice with positive metastatic lesion were not found to have detectable CTC, likelihood due to very few number or short-life time of CTCs existing either prior to initial CTC detection on Day 14, or beyond the scheduled time intervals for CTC detection, suggesting that the initial CTC detection could be started less than 2 weeks since the date of Sub-Q implantation, and more frequent periodical CTC detection should be performed during the entire study.

In accordance with our previous observation demonstrating that triploid and tetraploid CTCs respectively had intrinsic and acquired resistance to cisplatin in gastric cancer patients [16], *in situ* PK CTC performed by CK-i•FISH^®^ in this study was able to specifically pinpoint the distinct CTC subtypes either chemosensitive or chemoinsensitive. In cisplatin treated mice, all the CK18-/diploid and majority of CK18+/diploid CTC subtypes were cisplatin-sensitive and eliminated prior to Day 62. Among the 8 chemoinsensitive CTCs detected on Day 62, higher percentage (63%) of CK18+/multiploid cells were identified.. No recurrent CTC was detected in GA0087 mice as long as they were eliminated.

An interesting question arises from this study - how does cisplatin impact on CTC numbers in GA0087 PDX mice? Cisplatin, an alkylating-like chemotherapeutic agent, cross-links DNA of cancer cells, followed by triggering apoptosis and necrosis to those neoplastic cells. The heterogeneous population of CTCs consists of epithelial cells, EMT transition cells, hybrid epithelial/EMT tumor cells, irreversible EMT cells and circulating tumor stem cell (CTSC) [32]. In cancer patients, “mesenchymal CTCs” (epithelial marker CK/E-cadherin negative, mesenchymal marker vimentin/N-cadherin positive) are chemoresistant [20, 33, 34], which keeps in agreement with our previous report indicating the majority of gastric cancer CTCs were CK18- and chemoresistant [16]. In contrast to negative CK18 expression in majority of patients' CTCs, *in situ* PK CTCs performed by CK-i•FISH^®^ showed that most of the CTCs in various PDX models (Figure 6B) including GA0087 were CK18+, and many of them were eliminated following cisplatin treatment, suggesting that most of the CTCs in GA0087 PDX mice might be chemosensitive “Epithelial CTCs”. Several intriguing questions remain to be further investigated with respect to exploring how cisplatin impacts on decreasing CTC numbers in PDX mice, such as whether PDX CTCs are mesenchymal cells similar to that in patients; whether the EMT mechanism in PDX mice is similar to that of human; whether there is a correlation of CTC's ploidy and vimentin/CK18 expression with chemoresistance in PDX mice; and whether cisplatin targets only on PDX CTCs in blood, or exclusively on the cancer cells in primary solid tumor (Figure 3A), subsequently resulting in hematogenous dissemination of much fewer cancer cells, etc. Answering to those questions will help us understand more about the mechanisms of CTC's chemoresistance or sensitivity in both cancer patients as well as PDX tumor models.

In conclusion, obtained results in this study indicated that CTC can serve as an effective indicator for pre-clinical evaluation of anti-cancer agents efficacy in the metastatic PDX (mPDX) model. In addition to conventional enumerating metastatic nodules in post-mortem mice at the completion of the experiment, the more significant cellular and molecular strategy of dynamic monitoring CTC subtypes is able to constantly quantitate and characterize various hematogenous disseminated cancer cells following administration of therapeutic agents in pre-mortem mice in real time. Moreover, therapeutic agent sensitive- or resistant-tumorigenic polyclonal CTCs [28] or the feasible subcloned monoclonal CTC subtype derived xenograft mouse models (CDXs) could be a valuable alternative in addition to the conventional PDX model. *in situ* PK CTC by i•FISH^®^ is able to efficiently identify CTC subtypes possessing diverse chemosensitivities in both cancer patients and tumor animal models for further genetic and functional analyses performed on either pooled or single CTC [27, 35], and such unique advantage will help promote developing new anti-cancer drugs as well as cancer research.

## MATERIALS AND METHODS

### Establishment and genetic characterization of the metastatic HuPrime^®^ PDX model GA0087

Protocol of establishment of PDX model GA0087 was essentially similar to that previously published by us [36, 37]. Briefly, tumor tissue was surgically removed from a treatment-naive 74 years old female Asian patient diagnosed with gastric neuroendocrine tumor (ulcerative type, T3N1M0, TNM Stage II). Freshly dissected tumor tissue (3×3×3 mm^3^) was subcutaneously (Sub-Q) engrafted on the flank of 3-5 weeks old female immunocompromised Balb/c nude mice. When the size of tumor in the established primary tumor models (P0) reached 500-700 mm^3^ (1/2×L×W^2^), primary tumors were harvested and equally cut to the small fragments of 3×3×3 mm^3^, and subcutaneously re-engrafted on the flank of 3-5 weeks old female immunocompromised Balb/c nude mice for expansion (P1). After 3 consecutive passages, the xenograft was stabilized and subsequently subjected to model characterization, including histopathological analysis and gene expression microarray assay. Fresh tumor fragments at passage P3-P5 were frozen with 20% FCS in liquid nitrogen for further model recovery. Serial re-engrafting was repeated for no more than 10 times (P10). All procedures were sterilely performed at the SPF facility (Crown Bioscience, Santa Clara, CA, USA)

For profiling of gene expression in GA0087 model, fresh tumor tissues were dissected from GA0087 tumor-bearing mice, and subsequently snap-frozen at −80°C. Total RNA was isolated from the frozen tissues using Trizol according to the manufacturer's instruction (Life Technologies, Carlsbad, CA, USA), and purified by means of a RNeasy mini column (Qiagen, Valencia, CA, USA). Obtained RNA was subjected to a Bioanalyzer (Model 2100, Agilent Technologies, Santa Clara, CA, USA) for assessment of RNA quality. Only the high quality of RNA samples with RNA Integrity Number (RIN) ≥ 8 were applied for expression profiling assays on HG-U219 array plates (Affymetrix, Santa Clara, CA, USA). Raw CEL data sets of all samples were normalized by RMA algorithm according to the Affymetrix's protocol. Probe set intensity was expressed as log2 transformed values.

Clinical procedure applied on the patient in this study was approved by both the Institutional Review Boards of the Hebei Medical University Affiliated 4th Hospital (Hebei, China) and the informed consents from the patient. Entire animal study was conducted in accordance with the Guide for the Care and Use of Laboratory Animals of the National Institutes of Health. The protocol for animal study was approved by the Committee of the Ethics of Animal Experiments at Crown Bioscience, Inc. (Crown Bioscience IACUC Committee).

### Anti-tumor activity study

GA0087 PDX mice (P8) with tumor mass of approximate 150 mm^3^ were randomly divided into 2 groups (8 mice per group), followed by intravenously (IV) injecting with either 0.9% NaCl (vehicle) or cisplatin (4 mg/kg, Jiutai Pharmaceutical, Jinan, China) on Day 27 post Sub-Q on Day 1. Cisplatin was prepared at 0.4 mg/mL in 0.9% NaCl for injection. Administration of cisplatin or 0.9% NaCl was performed for total of 5 times at the indicated time intervals. Tumor volume (1/2×L×W^2^) of each mouse was measured twice weekly using a caliber. T/C value (percent of ΔT/ΔC), the read out of tumor response to the treatment, was calculated as tumor volume change between the final and initial measurement date in cisplatin group/tumor volume change in vehicle group. All the animals were sacrificed at the end of the experiment on Day 76. Both vehicle and cisplatin treated group respectively had 2 tumor free mice as negative control.

### Histopathological examination of the tumor metastatic lesion in lung

Lung tissues from all of the sacrificed GA0087 PDX mice were dissected and fixed in 10% neutral buffered formalin at 4°C for 24 hours, followed by tissue processing overnight. Processed tissues were subsequently embedded in paraffin. After deparaffinization and rehydration, tissue sections (16 μm in thickness) were treated with 0.01 M sodium citrate (pH 6.0) at 95ºC for 30 min, followed by staining with hematoxilyn and eosin (H&E). Number of tumor metastatic nodules in lung was enumerated by means of a bright-field microscope.

### Detection and characterization of CTC subtypes and CTM by SE-i•FISH^®^ in PDX mice

Blind detection and characterization of CTC subtypes by SE-iFISH in coded mouse blood samples were performed according to the product manufacture's instruction (Cytelligen, San Diego, CA, USA). Decoding, analysis and evaluation of CTC subtypes correlating to the animal tumor status were co-performed by cross blinded research scientists.

Retro-orbital bleeding (200 μl/mouse) was performed from Day 14 post-Sub-Q and periodically repeated at the indicated time intervals. For each bleeding, 200 μl of the collected mouse blood were immediately and thoroughly mixed with anti-mouse blood coagulant (Cytelligen) by gentle shaking. Samples were kept at 4ºC for up to 24 hours, followed by subtraction enrichment (SE) and CK-iFISH. Briefly, mouse blood samples containing anti-coagulant were incubated with anti-mouse leukocyte monoclonal antibodies conjugated to magnetic beads for 30 min at room temperature with gentle rotation. The mixed solution was loaded on the non-hematopoietic cell separation matrix and centrifuged at 400 x g for 5 minutes. Magnetic beads in collected supernatants were removed using a magnetic stand (Promega, Madison, Wisconsin, USA). CTCs in the solution were spun down at 1050 × g for 5 min in a micro-centrifuge tube. Cell pellet was applied on the coated CTC slide (Cytelligen) and air dried. For subsequent CK-iFISH, dried sample on the slide was incubated with Vysis Centromere Probe (CEP) 8 SpectrumRed (Abbott Laboratories, Abbott Park, IL, USA) using a S500 StatSpin ThermoBrite Slide Hybridization/Denaturation System (Abbott Molecular, Des Plaines, IL, USA) for 2 hrs, followed by incubation with monoclonal anti-human CK18 conjugated to Alexa Fluor 488 (Life Technologies) at room temperature for 1 hr [38, 39]. The sample was subsequently subjected to collection and analysis of CTC images acquired using a fluorescence microscope (Nikon, Model Ni-U) equipped with an appropriate filter set. Identified CTC was defined as DAPI+, CEP8+, CK18+/or -.

### Statistical analysis

Statistical analysis was similar to that previously published [40]. Nonparametric Mann-Whitney U test was applied to compare enumerated metastatic nodules and CTCs in GA0087 PDX mice of vehicle *vs* cisplatin treated group. Correlation analysis was performed by Spearman Rank Correlation test. Statistical analysis of all the data was performed with SPSS V18.0 software (SPSS Inc., Chicago, IL, USA). Survival (Time-to-Event) was analyzed by GraphPad Prism 5 (GraphPad Software, San Diego, CA, USA) which computes log-rank (Mantel-Haenszel) test. *P* < 0.05 was statistically significant, and *P* < 0.01 was statistically very significant. All the *P* values were double-sided.
